# Length of Utterance, in Morphemes or in Words?: MLU3-w, a Reliable Measure of Language Development in Early Basque

**DOI:** 10.3389/fpsyg.2017.02265

**Published:** 2018-01-08

**Authors:** Maria-José Ezeizabarrena, Iñaki Garcia Fernandez

**Affiliations:** ^1^Department of Linguistics and Basque Studies, Faculty of Arts, University of the Basque Country, Vitoria/Gasteiz, Spain; ^2^Department of Social Psychology and Methodology of Behavioral Sciences, Faculty of Psychology, University of the Basque Country, Donostia-San Sebastian, Spain

**Keywords:** MLU, Basque language, early language development, bilingualism, complexity

## Abstract

The mean length of utterace (MLU), which was proposed by Brown (1973) as a better index for language development in children than age, has been regularly reported in case studies as well as in cross-sectional studies on early spontaneous language production. Despite the reliability of MLU as a measure of (morpho-)syntactic development having been called into question, its extensive use in language acquisition studies highlights its utility not only for intra- and inter-individual comparison in monolingual language acquisition, but also for cross-linguistic assessment and comparison of bilinguals' early language development (Müller, 1993; Yip and Matthews, 2006; Meisel, 2011). An additional issue concerns whether MLU should be measured in words (MLU-w) or morphemes (MLU-m), the latter option being the most difficult to gauge, since new challenges have arisen regarding how to count zero morphemes, suppletive and fused morphemes. The different criteria have consequences, especially when comparing development in languages with diverging morphological complexity. A variant of MLU, the MLU3, which is calculated out of the three longest sentences produced (MLU3-w and MLU3-m), is included among the subscales of expressive language development in CDI parental reports (Fenson et al., 1993, 2007). The aim of the study is to investigate the consistency and utility of MLU3-w and MLU3-m as a measure for (morpho-)syntactic development in Basque, an agglutinative language. To that end, cross-sectional data were obtained using either the Basque CDI-2 instrument (16- to 30-month-olds) or the Basque CDI-3 (30- to 50-month-olds). The results of analyzing reports on over 1,200 children show three main findings. First, MLU3-w and MLU3-m can report equally well on very young children's development. Second, the strong correlations found between MLU3 and expressive vocabulary in the Basque CDI-2 and CDI-3 instruments, as well as between MLU3 and both nominal and verbal morphology scales, confirm the consistency not only of MLU3 but also of the two Basque CDI instruments. Finally, both MLU3-w and MLU3-m subscales appear sensitive to input after age 2, which emphasizes their utility for identifying developmental patterns in Basque bilinguals.

## Introduction

### Mean length of utterance: MLU and MLU3

How to measure language complexity is a question that has occupied linguists in a longstanding debate. Some authors maintain that since all languages are learnable by any child, they must have the same degree of complexity. In this regard, cross-linguistic differences found in complexity in each language component are believed to be the result of a compensation system, so that languages showing very high complexity in one particular domain are expected to have less complexity in other domains and vice-versa. In addition, the observation that, synchronically, many languages with low complexity in morphology have a rigid word order or a more complex phonological system than languages with complex morphology may support that assumption. However, counter-evidence has also been provided by scholars denying any theory-internal reason to predict similar degrees of complexity in all natural languages. See Newmeyer (2017) and Newmeyer and Preston (2014) for an overview of the debate.

The issue of language complexity piqued early language acquisition researchers' interest already in the beginning of the twentieth century. Such is the case of, for example, Nice (1925), who regarded *average sentence length* as “the most important single criterion for judging a child's progress in the attainment of adult language” (Rice et al., 2010). In a similar vein, five decades later, Roger Brown passionately defended his *Mean Length of Utterance* or *MLU*, which proved to be one of the most commonly-mentioned indexes of constructional complexity in child language by the end of the century:

“… *The MLU is an excellent simple index of grammatical development because almost every new kind of knowledge increases length: the number of semantic roles expressed in a sentence, the addition of obligatory morphemes, coding modulation of meaning […]and, of course, embedding and coordinating. All alike have the common effect on the surface form of the sentence of increasing length (especially if measured in morphemes, which includes bound forms like inflections rather than words)”* (Brown, 1973, pp. 53–54).

Brown considered MLU to be a more suitable index than age to compare individuals' development, since it permits identifying “on internal grounds” children who are “*at the same level of constructional complexity”* but who may not be “*of the same chronological age”* (Brown, 1973, p. 55).

In addition to the MLU calculated from the sentence sample uttered in a recording session, Brown regarded the upper bound or the longest sentence produced at a specific age as a relevant additional index to measure the attained grammar complexity of children. Thus, he established a sequence of five stages in children's earliest morphosyntactic development based on the two indexes: MLU and *upper bound*. Both values increased with age in the three longitudinal corpora analyzed (Eve, Adam, and Sarah). Each stage was associated with the child's productive use (at least in 90% of the contexts in which they are required) of some linguistic structures, and individual differences were observed in the age at which each child reached the various stages. For instance, Eve attained stage V at 2;2 years, whilst at that age Adam's and Sarah's MLU values around 2 indicated stage II. In Table 1 we have combined data which Brown presented separately: the target values of MLU and upper bound corresponding to each stage and the age ranges of the three children studied longitudinally at the different stages. The variability in age is evidenced by the large age ranges across stages displayed in column 4.

**Table 1:** Target values and approximations attained for MLU and upper bounds.

**Stage**	**Target value**		**Age in months of Adam, Eve, and Sarah**
	**MLU**	**Upper bound**	
I Semantic Roles and Syntactic Relations	1.75	5	18–30
II Grammatical Morphemes and the Modulation of Meaning	2.25	7	19–34
III Modalities of the Simple Sentence	2.75	9	20–36
IV Embedding	3.50	11	22–42
V	4.00	13	24–48

*Brown (1973, pp. 56–57)*.

Despite the advantages of an index other than age to compare children's linguistic development, Brown still pointed out some limitations, starting from Stage V onwards. He argued that, at that stage, children's varied linguistic productions and their MLU begin to depend more on the nature of the interaction than on what children know (Brown, 1973, p. 54).

Brown's view of complexity is not related to any specific language component such as semantics or morphology. It is based on the assumption that the acquisition of components such as *x* and *y* alone does not immediately, or even relatively quickly, lead to the acquisition of the construction *x* + *y* that combines the two. Consequently, in his *cummulative* sense of complexity, “*construction x* + *y* may be regarded as more complex than *x* or *y* because it involves everything involved in either of the constructions alone, plus something more” (Brown, 1973, p. 400). This lack of precision is probably what led researchers to question MLU's appropriateness to measure *morphosyntactic development*. Bickerton (1991), for instance, suggested that qualitative aspects of syntactic development cannot be directly evaluated, since the increase in length of utterances does not necessarily imply an increase in syntactic complexity. In fact, similar or higher MLU values (1a-c) may correspond to utterances with a lower morphosyntactic complexity, which is the case with the coordinated structures in (1a) as compared to S-V agreement examples in (1b) or the embedding structures in (1c).

(1) a. *Peter and Mary* (3 w / 3 m)
     b. *Ann comes* (2 w / 3 m)
     c. *want to come* (3 w / 3 m)

Thus, MLU may appear to be a quantitative rather than a qualitative measurement: “as utterances get longer and MLU increases, some sort of increase in complexity is bound to occur, but there is no *a priori* reason why the increase should take only the forms it does, and, in particular, that these forms should be the same for all children studied, whatever the language in question” (Brown, 1973, pp. 64–65). Additionally, issues such as how to measure children's achieved linguistic complexity and whether the same degree of complexity should be assumed at a particular stage cross-linguistically or across individuals acquiring a particular language have not received a convincing and generally accepted answer yet.

However, the generalized acquisition order of 14 inflectional markers in English established by Brown, which was confirmed in later longitudinal studies, reinforces the supposition of some pattern in morphosyntactic development which goes beyond the aforementioned individual variability. Despite MLU being originally “invented for English,” Brown was still aware of its utility in other languages for cross-linguistic comparison, once some adjustments were made: “Studies of highly inflected languages […], all report some difficulty in adapting our rules of calculation, invented for English, which is minimally inflected, to their languages. What I have used is, in each case, the author's choice of the linguistically most reasonable value” (Brown, 1973, p. 68). Actually, many longitudinal case studies conducted in typologically distant languages have provided relevant results regarding the specific structures which arise in children's spontaneous production at each specific developmental stage. Besides, MLU has been used in cross-sectional studies comparing early bilingual children's development in their two languages (Marchman et al., 2004; Meisel, 2011; Thordardottir, 2011; Hoff et al., 2014) as well as typical vs. atypical language development (Johnston, 2001; Rice et al., 2010; Wieczorek, 2010).

In his seminal 1973 book, Brown devoted part of the introductory section to describing and discussing the set of rules for calculating MLU and upper bound in spontaneous production corpora. Here are the most relevant ones: (a) a subsample is required to calculate MLU in a longer sample gathered at some specific developmental stage. However, not every utterance can be equally reliable in the sample: 100 utterances should be taken from the fully transcribed utterances, starting at the second transcription page rather than from the first minutes of the conversation; (b) stuttering or repeated attempts to produce some words or utterances are counted once, in the most complete form used. This rule may avoid under-scoring due to the selection of non-representative items of the child's (real) linguistic performance in constructional complexity; (c) fillers such as *umm* are not counted, in contrast to *no, yeah, hi*, which are included in the counting; (d) inflectional morphemes (plural, genitive, 3rd singular present –*s*, and so on) are counted as separate morphemes and inflected auxiliaries are counted as mono-morphemic words, as are compounds, for example, *birthday*. In our opinion, such counting criteria appear as an intermediate option between counting words and morphemes. However, such a counting system, together with the specific properties of English morphosyntax (a limited inventory of inflectional person and plural markers, low word complexity) and the scarcity of inflectional markers in children's early productions, may lead one to predict no great difference in measuring English child utterance length in words or in morphemes. In contrast, in languages with a certain degree of morphological complexity, like Basque, many researchers are in favor of measuring morphosyntactic development in morphemes rather than in words (Idiazabal, 1991; Barreña, 1995; Ezeizabarrena, 1996; Elosegi, 1998; Larrañaga, 2000; Larrañaga and Guijarro-Fuentes, 2012a). Nonetheless, the high (almost perfect) intralinguistic correlations between the two ways of calculating MLU found in such typologically distant languages as Spanish (Aguado, 1995; Jackson-Maldonado and Conboy, 2007), Irish, Icelandic and Dutch (see Parker and Brorson, 2005 and references therein), indicates that MLU-m may not necessarily be a better measurement than MLU-w. In contrast to authors who have suggested the higher usefulness of MLU-w because of the ease of calculating it, Wieczorek (2010) has questioned the fact that MLU-w and MLU-m can be regarded as similar indicators of morphosyntactic development simply because of the high correlations attested cross-linguistically. According to this researcher, MLU-w is related to lexical development rather than to grammatical development and therefore, the opposite is expected to be the case for MLU-m, which should show a stronger relation to grammatical rather than lexical development. A third way of calculating MLU in syllables (MLU-s) has also been explored in Irish (Hickey, 1991) and in Inuktitut (Allen and Dench, 2015). Surprisingly, MLU-s, which *a priori* would not be considered an index of grammatical development *per se*, or at least not in every language, also correlates with the previous indexes. The high correlations attested across languages between the different types of MLU may indirectly cast doubt on the “equivalence” of all of them as measures of language development, although determining exactly what the different variants of MLU measure in each language goes far beyond the aim of the current study.

Apart from the several ways of counting MLU, another objection to the use of MLU is the subjectivity present throughout the different steps preceding its calculation. To start with, MLU is sensitive to event and exchange patterns, situational variability and conversational dominance in a bilingual child, which may cause the sample collection on a particular date or conversational situation not to be the best example of the child's regular linguistic use (see Johnston, 2001 and references therein). Thus, counting all the sentences in a session or selecting the (50?, 100?, more?) utterances from the first, intermediate or final part of a two-hour recorded conversation may result in a different MLU value of a child's production at a particular age. Moreover, criteria for calculating MLU vary across studies, such as in the case of *MLU* vs. *alternate MLU* measures (Johnston, 2001), or of measuring MLU in words (MLU-w), morphemes (MLU-m) or syllables (MLU-s). Finally, subjectivity is present in the process of transcribing and coding oral speech in general, a task which “relies on the accuracy of the transcriber” (Rollins et al., 1996) and in the process of segmenting utterances. Segmenting words and especially morphemes in an utterance arises as the next complication in the process, where decisions regarding null morphemes, multimorphemic words such as portmanteaux, compounds and so on need to be made before starting with the analysis. Otherwise the variability found in children's spontaneous productions may lead to quite diverging value assignments to the same utterance. In order to regulate the subjectivity inherent in the processes mentioned above, single *individuals are* put *in charge* of the segmentation task of a whole set of recordings or of a sample collection, and further *interjudge reliability* rates are established on their codifications.

Despite the objections discussed earlier, MLU has still been extensively used in both intra- and inter-individual comparative studies. This is the case of, for instance, studies on language dominance which compare bilinguals' development in their two languages. On the assumption that length of utterances across languages may vary more depending on the unit in which its calculation is based, MLU-m has been proposed as a better measure for bilinguals' individual interlinguistic comparison in language pairs such as Basque-Spanish (Meisel, 1994; Ezeizabarrena, 1996; Elosegi, 1998; Larrañaga, 2000; Larrañaga and Guijarro-Fuentes, 2012a etc.), whilst studies on French-German bilinguals (Meisel, 1991; Müller, 1993; Müller and Kupisch, 2003; Kupisch, 2008; Schmeiser et al., 2016) or English-Mandarin bilinguals (Yip and Matthews, 2006) and even some on Spanish-Basque (Larrañaga and Guijarro-Fuentes, 2012b) have opted for MLU-w. See also Hickey (1991), who considers that MLU's utility for cross-linguistic comparison cannot be generalized even intraindividually.

Despite criticisms, MLU, in its different modalities, remains as one very relevant index for morphosyntactic development in longitudinal corpora of spontaneous language production, and the inclusion of some versions of it in assessment instruments confirms this fact. Such is the case of *MLU3*, included in the MacArthur-Bates Communicative Development Inventories (CDI) instrument (Fenson et al., 1993, 2007), a parental questionnaire designed to obtain normative data which may allow researchers to assess both typically and atypically developing children. The MLU3 is a combination of two indexes on which Brown's 5-stage classification was based (*mean length of utterance* and the *upper bound*). Yet MLU3 has the particularity that the mean length is calculated based on the child's three longest recently-produced sentences according to their parents, instead of on a specific sample of child utterances gauged by a researcher in a longitudinal corpus.

Studies on early bilingualism using this measurement have concluded that MLU3 values are sensitive to the amount of a child's exposure to the language. Bilinguals, who by definition have less exposure to their language(s) than monolinguals, have shown lower values than their age-matched monolingual counterparts (1;10–2;6: Hoff et al., 2012, 2014). More specifically, the results from Spanish-English bilingual groups, which were distinguished according to their higher, balanced and lower exposure to the language, revealed that the less input bilinguals had received in the language under study, the lower the scores they obtained in MLU3 values (Hoff et al., 2012).

### Utterance length in basque

From the genetic point of view, Basque is unrelated to any other known language; that is, it is an isolate language. Typologically, Basque is a null subject, ergative language with non-rigid SOV word order, a language with very rich nominal and verbal inflection (case marking, person and number subject-, direct object- and indirect object-agreement marking in the verb), with a predominantly agglutinative morphology and affixed postpositions. As a result, most nominal and verbal words comprise two or more morphemes (2a-c), which makes utterance length diverge, depending on whether it is measured in words (1,1 and 4 w) or morphemes (2, 4, and 8 m) in (2a), (2b) and (2c), respectively.

(2) a. *panpin-a*
          doll-Det   (1 w, 2 m)
          ‘doll’ or ‘the doll’
     b. *panpin-txo-a-rekin*
          doll-DIM-Det-with  (1 w, 4 m)
         ‘with the dolly’
      c. *Jon panpin-txo-a-rekin   etorri-ko   da*
          Jon doll-DIM-the-with      come-FU   Aux.S3s (4 w, 8 m)
          ‘Jon will come with the dolly”

However, not all morphemes are counted as productive morphology in early child productions. Following Brown's (1973) proposal of counting productive (non-rote learned) words and morphemes and taking into account both the specific morphosyntactic properties, as well as the characteristics of earliest productions in Basque, Idiazabal (1991) established the first list of rules to calculate MLU-m in Basque, which were followed in later longitudinal case studies (Barreña, 1995; Ezeizabarrena, 1996; Elosegi, 1998; Almgren, 2000; Larrañaga, 2000). According to these rules, diminutive suffix –*txo* is not counted as a morpheme in very frequent diminutive words in child and child-directed speech such as *ama-txo* “mumm-y” and *aita-txo* “dadd-y” (1 w / 1 m) but, on the other hand, –*txo* is counted as a morpheme in the rest of the few remaining words that include it (2a-c). Moreover, the –Ø morpheme is not counted, and the –*a* ending, which is translated as Det(erminer) in the (2a, 2b) glosses, is not counted as a morpheme either. There are several reasons for not counting this –*a* ending, which is suffixed to the nominal phrase rather than to the noun, as a (productive) morpheme: (a) many lexical roots having an organic –*a* ending do not modify their phonology when the determiner –*a* is suffixed (*musika* “music/music-Det”), (b) overtly determined roots like *etxe-a* “house-Det” cannot always be considered as such, since they can be used to respond to the question, “how do you say… *house* in Basque?”, where no determined nouns are expected; and (c) in early child Basque the nominal -*a* ending acts as an unanalyzed word boundary, rather than as a grammatical element, as seen in examples like *bestea umea* instead of *beste umea* “other child,” attested in several longitudinal samples (Barreña and Ezeizabarrena, 1999).

### Sociolinguistic context

Basque is a language spoken in the North Eastern area of Spain and the South West area of France, on both sides of the Atlantic Pyrenean mountains. All adult speakers of that language are bilingual Spanish-Basque or French-Basque. The Basque-speaking community of roughly one million speakers mostly comprises people who grew up in Basque-speaking families and acquired Basque as their L1 (either simultaneously or alongside Spanish or French, successively) and early L2 speakers who, growing up in almost monolingual Spanish or French families, are exposed to Basque very early (from age 2 or 3 onwards) through the educational system. Another group of late L2 speakers acquired that language through adult training courses. Sociolinguistic surveys conducted in 2006 with population older than 15 years of age in the Basque Country described the following distribution of linguistic profiles: 15.4% passive bilinguals, 25.7% active bilinguals and 58.9% French or Spanish monolinguals. Further censal surveys conducted in the Basque Autonomous Community, the region in which most of the current sample was collected, concluded that 39% of the 5- to 9-year-old population had Basque exclusively or together with Spanish as their home language (Basque Government, 2009). Consequently, most L1 Basque-speaking children are exposed to different degrees of Spanish (or French) input, and this is also the case of the participants of our study.

### Aims and predictions

The current paper investigates MLU3 scales' reliability as compared to other scales of the Basque CDI to assess early language development in that agglutinative language. For that, it provides data of 16- to 50-month-old children obtained using the Basque versions of the MacArthur-Bates CDI parental questionnaires.

In a language community such as the Basque-speaking one, in which being bilingual is the norm rather than the exception, the assumption that monolingual data are the best reference for “typical development” does not hold, and consequently, only instruments which are sensitive to the amount of exposure to the language(s) can accurately assess early bilingual language development. Therefore, a further study conducted with a subsample of over 1200 18- to 48-month-olds' MLU3-w and MLU3-m scores will analyse those measurements' sensitivity to two variables, chronological age and (relative) amount of exposure to the Basque language, with the aim of checking MLU3 subscales' utility in that particular context. Three predictions can be stated in this regard:

1. MLU3 scales will be as sensitive as the rest of the scales in the Basque CDI instrument to detect children's developmental changes as found in previous studies, and will reflect development in morphological complexity (Fenson et al., 1993, 2007).
2. Taking into account the morphosyntactic properties of an agglutinative language with rich morphology, such as Basque, MLU3 measured in morphemes will prove to be more discriminative than the MLU3 measured in words.
3. Input quantity will affect children's expressive language. Hence, differences in length of utterance are expected among bilinguals, depending on children's relative amount of exposure to Basque, as widely reported in early bilingual research (Marchman et al., 2004; Meisel, 2011; Thordardottir, 2011; Hoff et al., 2014).

## Materials and methods

### Instruments

The MacArthur-Bates Communicative Development Inventory (CDI) instrument is a parental questionnaire used to gather information regarding children's language use. Different versions of the instrument have been developed, all designed for different age ranges (CDI-1 for 8–15 months, CDI-2 for 16–30 months, and CDI-3 for 30–50 months) and for different purposes such as screening (short CDI-1 and CDI-2) or clinical diagnosis and research (full CDI-1 and CDI-2 questionnaires) (see Fenson et al., 2000). The CDI-1 is the only instrument which includes vocabulary comprehension in addition to expressive vocabulary and grammar. In contrast, CDI-2 and CDI-3 are oriented to expressive language use.

The current study reports on data obtained with the long version of the CDI-2 and the CDI-3, for which there is only one (short) version. The Basque version of the full CDI-2 instrument (16–30 months), henceforth *BCDI-2*, contains different sections such as vocabulary and morphology, in which informants tick the items their child already produces, some questions about whether the child has started combining words, as well as a section for writing down the child's three longest recently-produced sentences. In addition, there is a list of multiple-choice items in which informants choose, from the different options the one that best fits with the child's current production. Filling in this questionnaire may take between 10′ and 60′, depending on the child's level of expressive use.

The Basque version of the CDI-3 instrument (30–50 months), henceforth the *BCDI-3*, is much shorter than the CDI-2. The BCDI-3 contains a vocabulary list, a grammar section, a section for writing down the three longest utterances, a list of multiple-choice items and a list of questions intended to assess children's knowledge of some logical and mathematical terms.

The sections and number of items analyzed in the current study are presented in Table 2. Neither the 37/29 items of the multiple-choice item section nor the 10 yes/no questions on logical concepts (included only in BCDI-3) have been included in the current analysis, since they are less homogeneous in format, across items and across the two instruments.

**Table 2:** Number of items in the BCDI scales included in the study.

	**BCDI-2**	**BCDI-3**
Vocabulary	643^a^	120
Nominal morphology	17	16
Verbal morphology	40	20
MLU3		

a *For the current study, some postpositions, included in the vocabulary section of the questionnaire were analyzed as morphological suffixes rather than as vocabulary items. Consequently, the distribution of (vocabulary/grammatical) items included in this study will vary from previous studies such as Barreña et al.'s (2008a,b), conducted with the same data sample*.

### Participants

The parents of over 2,000 children aged between 16 and 50 months of age participated in the study, filling in one of the two instruments: either the BCDI-2 (16–30 months) instrument (Barreña et al., 2008a) or the BCDI-3 (30–50 months) instrument (Garcia et al., 2014). The questionnaire is written exclusively in Basque. Consequently, all the informants in this study are bilingual parents with different levels of language use who interact in Basque and (at least) one other language on a daily basis and address their child (some exclusively, others mostly or only sometimes) in Basque. Participants gave informed consent prior to participation. The study was approved by the ethics commission of the University of the Basque Country.

**Figure 1:**
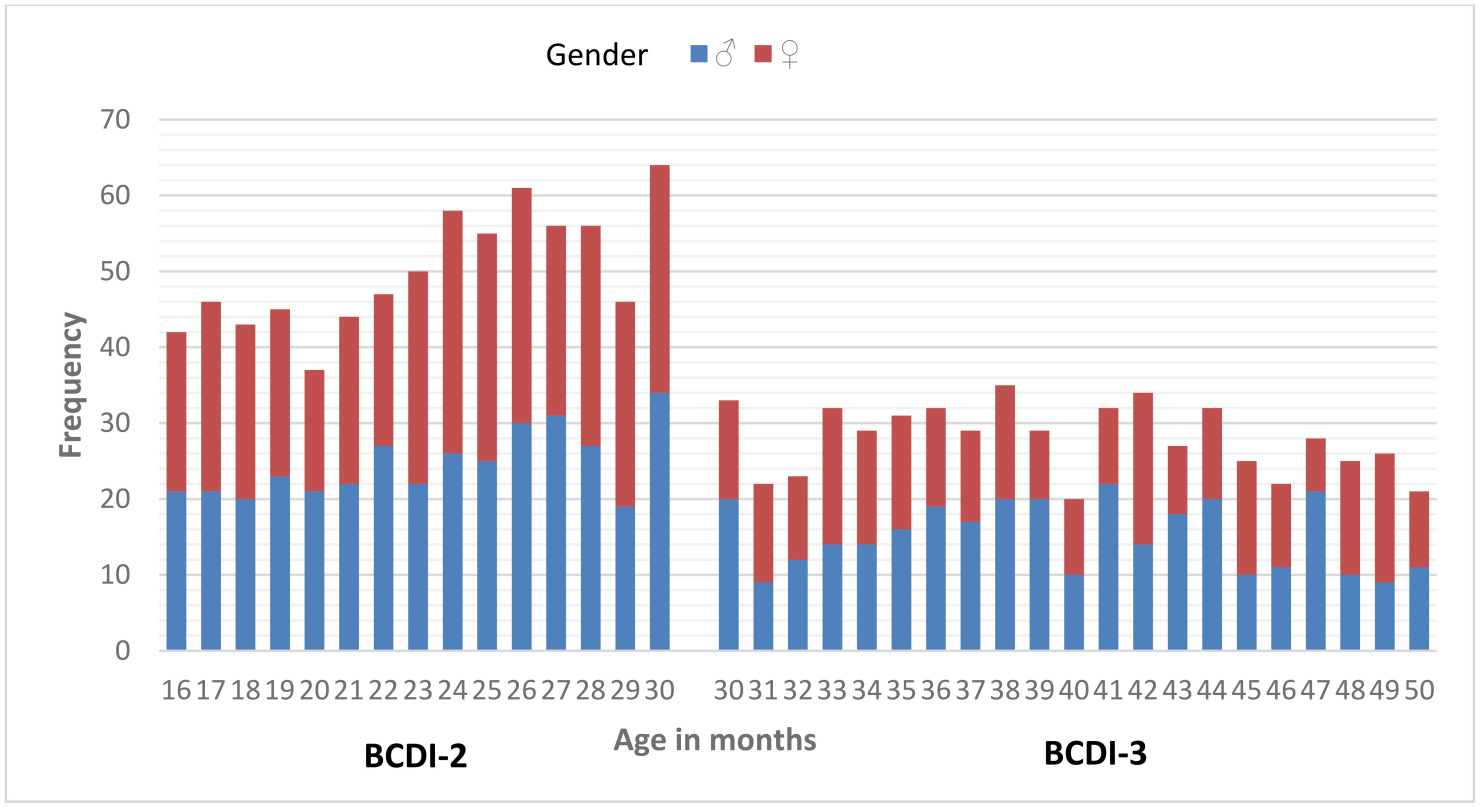
BCDI-2 and BCDI-3 sample by age and gender.

The data sampling lasted over a decade. The initial data collection of 2,248 questionnaires (BCDI-2 *n* = 1,204 / BCDI-3 *n* = 1,044) was filtered out based on a set of exclusion criteria: out of the age range (101 out of 15–30 months/26 older than 50 months), below 8-month-pregnancy pre-term born children (15/7), children who had over two ear infections during the first year (20/55); questionnaires in which vocabulary and/or grammar sections were incomplete (93/0) and questionnaires where any (one, two, or three) of the three longest utterances produced (207/389) and/or input data (25/15) were missing. Thus, the data sample of 16- to 50-month-olds analyzed for the current study includes 1,337 questionnaires (BCDI-2 *n* = 750/BCDI-3 *n* = 587). As shown in Figure 1, all age groups (in months) consist of a range of 20–64 participants for the whole period studied. As for gender, girls and boys are evenly distributed across the age groups [χ^2^_(14)_ = 6.27, *p* = 0.96 in BCDI-2 and $\chi_{\left(20\right)}^{2}$ = 28.18, *p* = 0.11 in BCDI-3].

In order to investigate the effect of input and age and the interaction between these two variables on MLU scores, the sample was limited to children aged between 1;6 and 4 years. The sub-sample of 1202 participants was divided into age groups and input groups. Five groups resulted from the division in six-month age groups (18–24 months, 25–30 months, 31–36 months, 37–42 months and 43–48 months). Each age group was further divided into four different input groups based on the relative amount of exposure to Basque and Spanish: Monolingual or M (over 90% Basque input), Basque-dominant bilingual or BDB (Basque input 60–90%), Balanced Bilingual or BB (Basque input 40–60%) and Spanish-dominant bilingual or SDB (below 40% Basque input) (see Table 3). In what follows, we will use the terms *input* or *relative input* to refer to the relative amount of exposure to Basque and Spanish, following Thordardottir (2011), among others.

**Table 3:** Distribution of the sample (raw numbers of participants and percentages) in age and input groups.

	**Age groups (range in months)**				
**Input groups**	**18–24 months**	**25–30 months**	**31–36 months**	**37–42 months**	**43–48 months**
Monolingual	200 (61.7%)	221 (59.6%)	107 63.3(%)	109 (60.9%)	102 (64.1%)
Basque-dominant bilingual	63 (19.4%)	96 (25.9%)	26 (15.4%)	26 (14.5%)	26 (16.4%)
Balanced bilingual	41 (12.6%)	35 (9.4%)	22 (13.0%)	20 (11.2%)	19 (11.9%)
Spanish-dominant bilingual	20 (6.2%)	19 (5.1%)	14 (8.3%)	24 (13.4%)	12 (7.6%)
Total	324 (100%)	371 (100%)	169 (100%)	179 (100%)	159 (100%)

### Procedure and coding

As in the original CDI, the grammar section of the BCDI includes several items regarding nominal inflection, verbal inflection and an item in which participants are requested to report on the child's longest three sentences produced recently. The MLU3 was calculated from the three utterances reported, as displayed in (3).

(3) Idatzi zure haurrak azken aldian esan dituen hiru esaldi luzeenak. ‘*Please write down the longest three sentences your child has recently produced’:*
  a. Ni-k  ur-a-Ø              nahi du-t     (4w 6m)
       I-Erg water-Det-Abs   want Aux.S1s.O3s
    a'. ‘I want water’ (3w 3m)
  b. Zu-Ø        kale-ra   joan-Ø z-ea    (4w 6m)
        you-Abs   street-to go       Aux.S2s
  b'. ‘you have gone/went to the street’ (5w, 6m)
  c. Unai-ren   bila          g-oa-z (3w 5m)
        Unai-Gen  look-for   go.S1pl
    c'. ‘we go looking-for Unai’ (4w, 4m)

Examples in (3) illustrate the three longest utterances of a 28-month-old child randomly chosen from the BCDI-2 sample and the way they were measured. Thus, MLU3 in (3) was calculated based on the mean of the length of the three utterances reported. So that MLU3-w of (3a + 3b + 3c) / 3 is (4 + 4 + 3) / 3, that is, 3.66 and MLU3-m is (6 + 6 + 5) / 3, namely, 5.66. This shows that MLU-w and MLU3-m differ considerably in Basque. In contrast, measuring utterance length in MLU-w or MLU-m in a language with predominantly monomorphemic words like English (3a', 3b', 3c') does not make much difference: MLU3-w: 12/3 = 4; MLU3-m = 13/3 = 4.33.

MLU3-w and MLU3-m calculations were performed by two independent coders. The high coefficients of intraclass correlation resulting from the statistical analysis for both MLU3 scales in the two instruments (*r* = 0.91 and α = 0.95 for MLU3-w; *r* = 0.94 and α = 0.96 for MLU3-m in BCDI-2; *r* = 0.95 and α = 0.97 for MLU3-w; *r* = 0.95 and α = 0.98 for MLU3-m in BCDI-3) confirmed an excellent interjugde reliability of the data (Koo and Li, 2016).

Only the children who had not started combining words yet (their parents responded with “not yet” to the item preceding the three longest utterance section) obtained *1* as a mean value for the two variables, MLU3-w and MLU3-m. The rest of the children obtained higher values.

The results from the MLU sections will be analyzed together with the scores obtained in three more scales: vocabulary, nominal inflection, and verb morphology. In the vocabulary and morphology sections, informants were asked to tick the items their child had started producing. The final score was calculated by summing up the total number of items ticked in each of the sections.

The maximal potential score in *vocabulary* was 643 items in BCDI-2 and 120 in BCDI-3. *MLU3-w* and *MLU3-m* were open scales and therefore no maximal values could be estimated *a priori*.

As for *nominal morphological markers*, 17 items from BCDI-2 and 14 items from BCDI-3 were analyzed for the current study and consequently, 17/14 were the highest possible scores in this section, respectively. The items analyzed from BCDI-2 are the following: 11 postpositional suffixes (*-n, -ra, -raino, -rantz, -tik, -zkoa, -koa, -z*, -*rena, -rentzat, -rekin*) and 6 non-postpositional ones (plural -*k*, genitive possessive -*ren*, genitive locative -*ko*, ergat *-k*, dative -*ri*, and diminutive -*txo*)^1^. BCDI-3 contains 11 postpositions (*-n, -ra, -raino, -tik, -zkoa, -koa, -z, -rena, -rentzat, -rekin, -rengna*) and 3 more nominal suffixes (plural -*k*, ergative -*k*, dative -*ri*).

As for *verbal inflection*, the maximal possible score was 39 in BCDI-2 and 22 in BCDI-3, corresponding to the number of items included in the two instruments in the current study. The items in BCDI-2 are three aspectual suffixes (imperfective -*tzen*, future -*ko*, and perfective -*ta*) in addition to 36 inflected frequent verb forms, most of them auxiliary forms. The items included in BCDI-3 (22) are two aspectual suffixes (imperfective -*tzen*, and future -*ko*) and 20 very frequent, most of them inflected auxiliary verb forms (*naiz* “am,” *da* “is,” *dago* “is”, *dizut* “I have…it to you,” *zenuen* “you had…it”).

### Data analysis

One-way ANOVAs were conducted separately for BCDI-1 and BCDI-2 instruments in order to measure the effect of age. In addition, Pearson's correlations were calculated to analyse between-scale relations, and finally, partial correlation coefficients were computed between BCDI scales with age as the covariate.

On the other hand, two-way ANOVAs were performed to compare the main effects of age and input in the whole sample, as well as the interaction between age and input in MLU3-w and MLU3-m scales. The effect size was calculated according to Cohen (1992) and Richardson (2011).

## Results

A variety of structures and morphological markers are attested in the sample of utterances produced by the participants, based on their parents' reports. The examples of 24-month-olds listed in (4a-b) and of 30-month-olds in (4c-d) were collected using the BCDI-2, whereas examples from 30-month-olds (4e-f), 36-month-olds (4f-h), 42-month olds (4i-j) and 48-month-olds (4k-l) were obtained using the BCDI-3 instrument. As expected in a language with rich case and inflectional morphology, length of utterance varies depending on whether it is measured in w(ords) or in m(orphemes) and the older the children become, the more complex are the structures attested. Thus, morphologically complex structures which are rare among children younger than 30 months, such as inflected verb forms with multiple agreement markers (4d), postpositional complex phrases (4f, 4h), embedded sentences carrying embedding particles (9g, 9k, 9l), start being reported from 2;6 and 3 years onwards or even later.

- amona    etxea-n   dago (cod. 628, 24 months: 3 w / 4 m)
  Grandma house-in   is
  ‘Grandma is at home’
- Josu-k     apurtu dau    (cod. 455, 24 months: 3 w/ 5 m)
  Josu-Erg. break   have.S3s
  ‘Josu has broken (something)’
- nahi   duzu        ni-rekin   jolastu kale-an? (cod. 481, 30 months: 5 w/ 8 m)
  want   have.S2s I-with      play    street-in
  ‘Do you want to play with me on the street?
- Ez   uz-ten           hau   egi-ten (cod. 1110, 30 months: 4 w/ 6 m)
  Neg  permit-ting   this   do-ing
  ‘not permitting doing this’
  Intended: *ez dit uzten hau egiten* ‘it does not permit me to do this’
- Ni  bakarrik  esnatu  naiz  (cod. 1041, 30 months, 4 w / 5 m)
  I    alone      wake    be.1s
  ‘I woke up alone’
- Amatxu   lan-era   joan   da   kotxe    barria-n (cod. 342, 30 months, 6 w/ 8 m)
  Mommy   work-to  go     is   car        new-in
  ‘Mommy went to work in the new car’
- Etxe-ra   etorri   n-aiz-enien    pelota-gaz jolastu do-t (cod. 1079, 36 months, 6 w/ 11 m)
  house-to come   be.S1s-when   ball-with play       have.S1s
  ‘when I came home I will play with the ball’
  Intended: *etxera etor-ten naizenien*…‘when I will come home…’
- Amatxi-ren   etxea-n   ardia   ikusi   dut     (cod. 7032, 36 months, 5 w / 8 m)
  Grandma-of   house-in sheep see     have.S1s
  ‘I have seen a sheep in grandma's house’
- zu   hemen  geratu-ko      z-ara   ni-rekin? (cod. 842, 36 months, 5 words, 8 morphemes)
  you here     leave-FUT    have.S2s me-with
  ‘will you stay here with me?’
- osaba-k     zergatik   ez   dauka   txabola Patxik        bezala? (cod. 566, 42 months, 7 w / 10 m)
  uncle-Erg why          Neg own.S3 cabin   Patxi-Erg   like
  ‘why does not the uncle have a cabin like Patxi has?’
- txikia   nintz-en-ean       sehaska-n   egi-ten nue-n lo (cod. 7040, 36 months, 6 w /12 m)
  small   be.1s-Past-when crib-in        do-IMP have.S1s-Past sleep
  ‘when I was that little I slept in the crib’
- Eskola-ko   jantokia-n      ema-ten   di-gu-te-n ogia      oso   goxoa   da   (cod. 7049, 48 months, 8 w / 14 m)
  School-of dining              room-in    give-IMP   have.S3pl.IO1pl bread very tasty is
  ‘the bread that they give us in the school meals is very tasty’
- gaur   Amaiur ez    da ikastola-ra etorri gaixorik dauelako (cod. 536, 48 months, 8 w / 10 m)
  today Amaiur Neg   is school-to come  sick is-because
  ‘today Amaiur did not come to school because he is sick’

### BCDI-2 (16-30 months)

**Figure 2:**
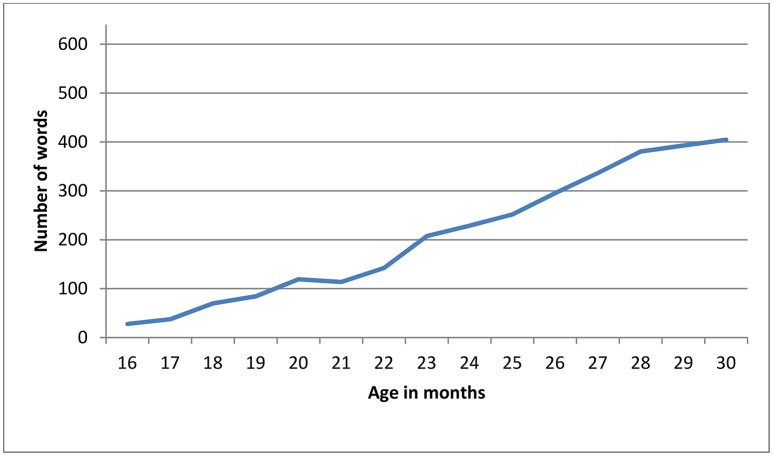
Mean vocabulary scores by age in BCDI-2 (643 items).

**Figure 3:**
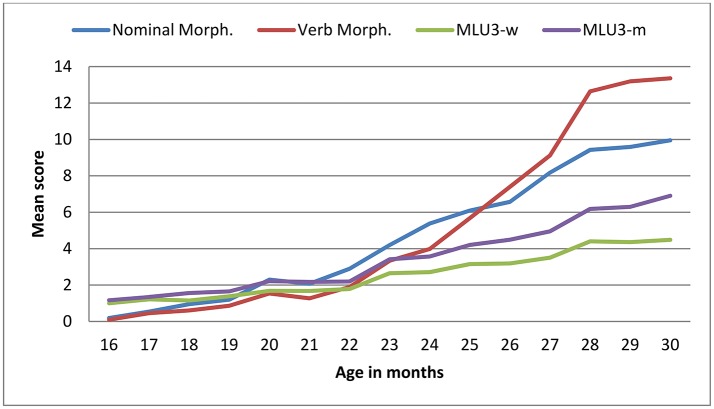
Mean scores by age in BCDI-2 scales: nominal morphology (17 items), verbal morphology (39 items) and MLU3.

The scores on all scales of the BCDI-2 increased significantly with age, as depicted in Figures 2, 3 (minimal-maximum scores: 0–643 in vocabulary, 0–17 in nominal morphology, 0–36 in verbal morphology, 1–10 in MLU-w and 1–16 in MLU-m). Mean and standard deviation values of BDCI-2 scales are shown in Table 4.

**Table 4:** Mean scores and standard deviations of five CDI-2 scales, by age in months.

		**Vocabulary**		**Nominal morphology**		**Verb morphology**		**MLU3-w**		**MLU3-m**	
**Age**	***N***	***M***	***SD***	***M***	***SD***	***M***	***SD***	***M***	***SD***	***M***	***SD***
16	42	27.93	34.47	0.19	0.55	0.09	0.30	1.00	0.00	1.17	0.38
17	46	37.43	65.83	0.54	2.00	0.46	2.24	1.21	0.91	1.35	1.19
18	43	69.81	80.54	0.95	2.22	0.60	1.80	1.15	0.51	1.57	0.85
19	45	84.29	114.19	1.20	2.59	0.87	3.34	1.38	0.63	1.65	0.84
20	37	119.27	120.92	2.30	4.07	1.54	3.21	1.68	0.93	2.21	1.33
21	44	113.64	101.89	2.07	2.96	1.27	2.00	1.67	0.94	2.17	1.30
22	47	142.36	123.81	2.89	3.93	1.89	2.96	1.78	1.03	2.20	1.26
23	50	207.44	149.54	4.20	4.26	3.34	4.44	2.65	1.54	3.41	2.23
24	58	228.64	116.43	5.38	4.41	3.98	4.19	2.71	1.35	3.57	1.94
25	55	251.84	139.46	6.09	4.85	5.67	5.86	3.15	1.62	4.20	2.33
26	61	295.29	131.48	6.57	4.46	7.39	6.85	3.19	1.51	4.49	2.36
27	56	336.37	137.64	8.18	4.50	9.12	7.13	3.51	1.75	4.95	2.64
28	56	380.48	151.64	9.43	4.89	12.64	8.98	4.40	1.81	6.18	2.87
29	46	392.72	153.11	9.59	4.99	13.20	9.10	4.36	1.99	6.30	3.44
30	64	404.50	160.59	9.95	5.09	13.36	9.53	4.49	1.95	6.91	3.27
Total	750	220.52	178.68	5.02	5.24	5.51	7.52	2.69	1.84	3.70	2.86

*Number of items by scale: vocabulary (643), nominal morphology (17), and verbal morphology (39)*.

The ANOVA analysis revealed a significant effect of age on all the scales of the BCDI-2: vocabulary [*F*_(14, 735)_ = 54.71, *p* < 0.001, $\eta_{p}^{2}$ = 0.51], nominal morphology [*F*_(14, 735)_ = 37.38, *p* < 0.001, $\eta_{p}^{2}$ = 0.42], verbal morphology [*F*_(14, 735)_ = 35.99, *p* < 0.001, $\eta_{p}^{2}$ = 0.41], MLU3-w [*F*_(14, 735)_ = 39.24, *p* < 0.001, $\eta_{p}^{2}$ = 0.43] and MLU3-m [*F*_(14, 735)_ = 40.20, *p* < 0.001, $\eta_{p}^{2}$ = 0.43]. Age effect on each scale was large according to Cohen (1992) and Richardson (2011).

As shown in Table 5, correlations between vocabulary, nominal morphology, verbal morphology, MLU3-w and MLU3-m scales were strong (*r* range: 0.81–0.97), especially between MLU3-w and MLU3-m (*r* = 0.97). Some correlation coefficients decreased after controlling for age, but their values remained both significant and high (*r* range: 0.66–0.95). Cronbach's alpha was 0.97 for the five scales^2^.

**Table 5:** Pearson's correlations between BCDI-2 scales (and partial correlations, controlling for age).

	**Nominal morphology**	**Verbal morphology**	**MLU3-w**	**MLU3-m**
Vocabulary	0.89 (0.80)	0.83 (0.71)	0.81 (0.66)	0.82 (0.68)
Nominal morphology		0.85 (0.76)	0.82 (0.70)	0.83 (0.72)
Verbal morphology			0.82 (0.70)	0.84 (0.74)
MLU3-w				0.97 (0.95)

*All correlations were significant at p < 0.001*.

### BCDI-3 (30–50 months)

**Figure 4:**
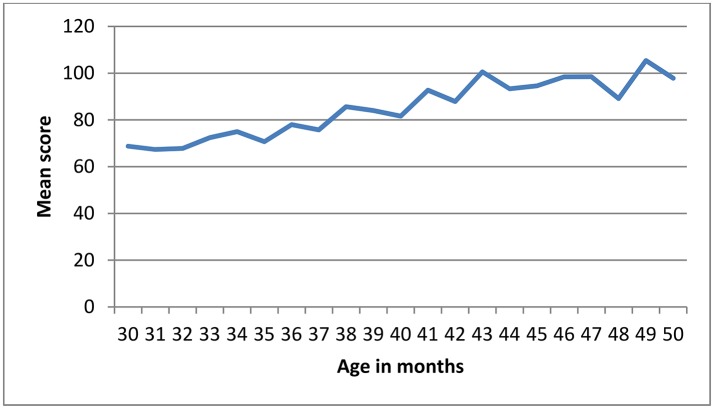
Mean vocabulary scores by age in BCDI-3 (120 items).

**Figure 5:**
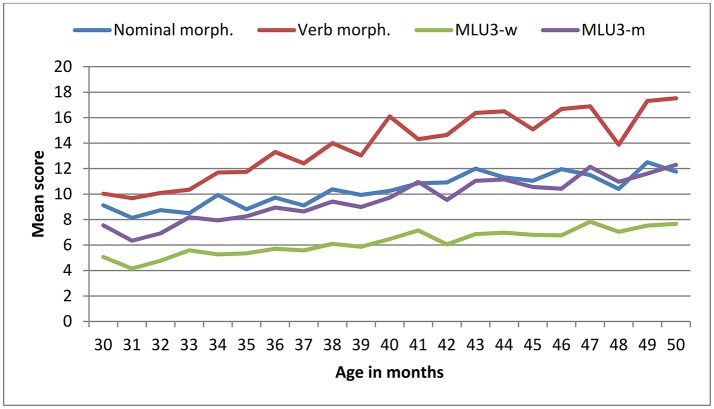
Mean scores by age in BCDI-3 scales: nominal morphology (14 items), verbal morphology (22 items) and MLU3.

The scores on all the BCDI-3 scales increased with age, as depicted in Figures 4, 5 and the effect size of age was large. Mean and standard deviation values of BDCI-3 scales are shown in Table 6.

**Table 6:** Mean scores (*M)* and standard deviations (*SD*) of five BCDI-3 scales by age in months.

		**Vocabulary**		**Nominal morphology**		**Verbal morphology**		**MLU3-w**		**MLU3-m**	
**Age**	***N***	***M***	***SD***	***M***	***SD***	***M***	***SD***	***M***	***SD***	***M***	***SD***
30	33	68.76	24.73	9.12	2.71	10.03	4.50	5.06	1.71	7.55	2.83
31	22	67.36	34.06	8.14	4.66	9.68	5.89	4.15	2.07	6.34	3.33
32	23	67.83	33.30	8.74	4.39	10.09	6.95	4.77	2.35	6.92	4.06
33	32	72.41	25.41	8.50	3.90	10.34	5.43	5.59	3.31	8.18	5.07
34	29	74.96	20.99	9.93	2.71	11.69	5.52	5.26	2.16	7.94	3.47
35	31	70.68	28.41	8.81	3.92	11.74	5.93	5.35	2.31	8.25	4.00
36	32	77.97	30.20	9.72	3.70	13.31	6.25	5.71	2.13	8.94	3.77
37	29	75.76	31.20	9.10	4.06	12.41	6.26	5.58	2.37	8.64	4.16
38	35	85.66	30.16	10.37	3.42	14.00	6.48	6.09	2.54	9.41	4.25
39	29	84.00	26.22	9.93	3.98	13.03	6.58	5.86	3.20	8.99	5.06
40	20	81.60	23.26	10.25	3.81	16.10	5.74	6.47	2.66	9.72	3.82
41	32	92.72	26.95	10.84	3.08	14.31	6.12	7.15	3.27	10.96	5.20
42	34	87.88	29.95	10.91	3.12	14.65	6.70	6.05	2.39	9.54	3.89
43	27	100.56	22.02	12.00	2.80	16.37	6.01	6.86	2.57	11.04	4.50
44	32	93.31	23.26	11.31	3.07	16.50	4.90	6.97	2.62	11.15	3.99
45	25	94.56	26.32	11.04	3.88	15.08	6.90	6.80	3.00	10.56	4.49
46	22	98.41	19.76	11.95	2.48	16.68	4.41	6.76	2.71	10.42	4.00
47	28	98.50	24.88	11.50	3.50	16.89	5.14	7.83	2.62	12.14	5.03
48	25	89.12	29.21	10.40	3.70	13.88	7.38	7.04	3.11	10.97	4.88
49	26	105.38	15.66	12.50	1.65	17.31	4.70	7.53	2.75	11.61	4.63
50	21	97.81	25.74	11.76	3.22	17.52	5.51	7.66	2.57	12.30	4.02
Total	587	84.69	28.67	10.29	3.61	13.79	6.32	6.19	2.74	9.56	4.49

*Number of items by scale: vocabulary (120), nominal morphology (14) and verbal morphology (22)*.

The ANOVA analyses revealed significant effects of age on all the BCDI-3 scales: vocabulary [*F*_(20, 566)_ = 5.46, *p* < 0.001, $\eta_{p}^{2}$ = 0.16], nominal morphology [*F*_(20, 566)_ = 3.56, *p* < 0.001, $\eta_{p}^{2}$ = 0.11], verbal morphology [*F*_(20, 566)_ = 5.03, *p* < 0.001, $\eta_{p}^{2}$ = 0.15], on MLU3-w [*F*_(20, 566)_ = 3.822, *p* < 0.001, $\eta_{p}^{2}$ = 0.12] and MLU3-m [*F*_(20, 566)_ = 4.14, *p* < 0.001, $\eta_{p}^{2}$ = 0.13].

A strong correlation was found across all the BCDI-3 scales, as displayed in Table 7: vocabulary, nominal morphology, verbal morphology, MLU3-w and MLU3-m (*r* range: 0.55–0.97). Again, the correlation between MLU3-w and MLU3-m was particularly high (*r* = 0.97). After controlling for age, some correlation coefficients decreased (*r* range: 0.51–0.97), but the values remained significant and high^3^. Cronbach's alpha was 0.91 for the five scales.

**Table 7:** Pearson's correlations between BCDI-3 scales (and partial correlations, controlling for age).

	**Nominal morphology**	**Verbal morphology**	**MLU3-w**	**MLU3-m**
Vocabulary	0.83 (0.81)	0.76 (0.73)	0.57 (0.51)	0.60 (0.54)
Nominal morphology		0.78 (0.76)	0.55 (0.51)	0.58 (0.54)
Verbal morphology			0.58 (0.52)	0.60 (0.55)
MLU3-w				0.97 (0.97)

*All correlations were significant at p < 0.001*.

### Input and MLU3

**Figure 6:**
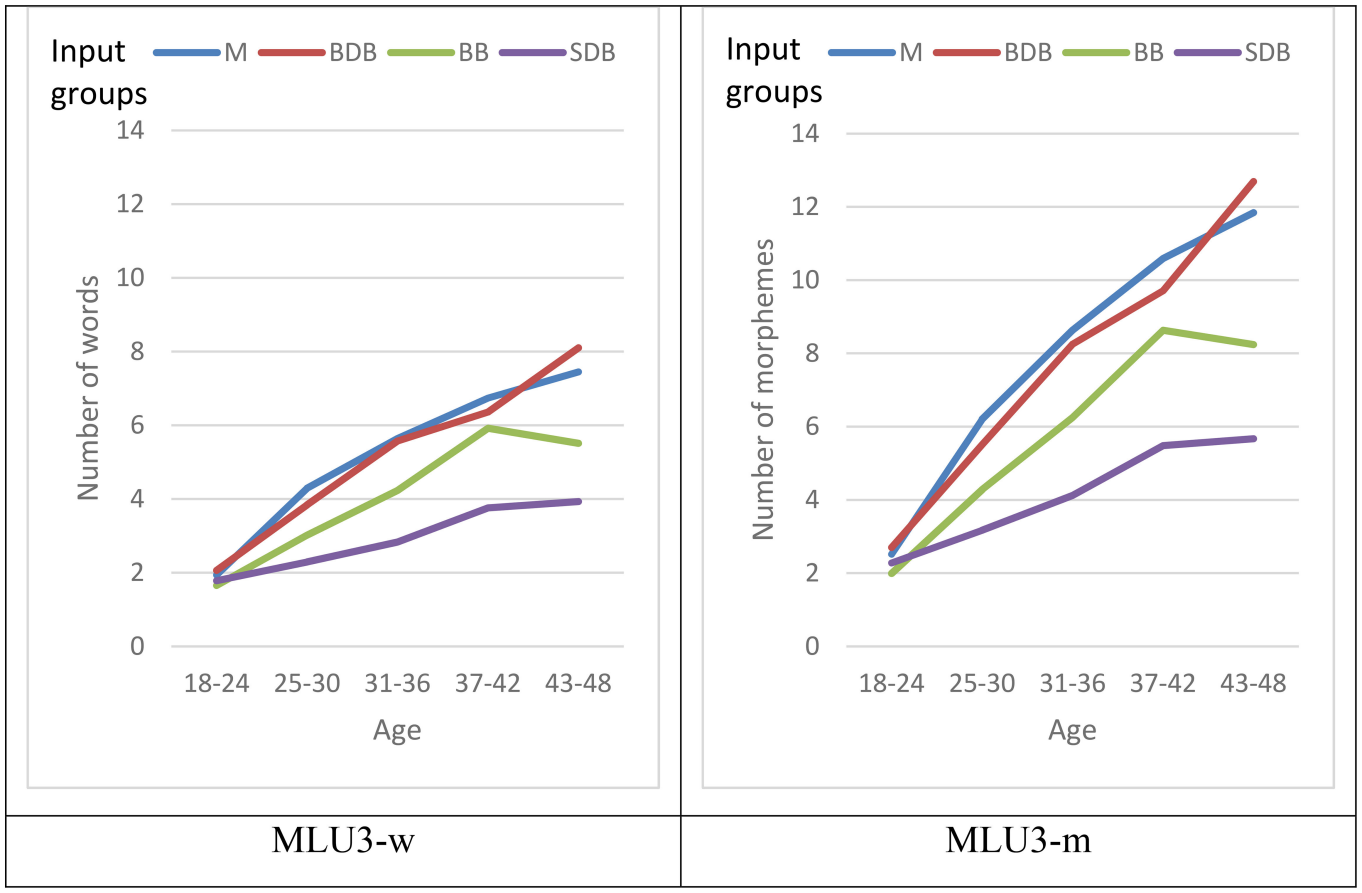
Means of MLU3-w and MLU3-m scores by age and input group: Monolingual (M), Basque-dominant bilingual (BDB) and Spanish-dominant bilingual (SDB).

Two-way ANOVA analyses were performed in order to investigate the effect of age, input (the relative amount of exposure to Basque and Spanish), and the interaction between them on the two MLU3 measures, MLU3-w and MLU3-m in the whole sample, which is depicted in Figure 6.

The first ANOVA showed main effects of both, age [*F*_(4, 1182)_ = 102.11, *p* < 0.001, $\eta_{p}^{2}$ = 0.26] and input [*F*_(3, 1182)_ = 41.01, *p* < 0.001, $\eta_{p}^{2}$ = 0.09] in MLU3-w and the interaction between these two variables yielded statistically significant results [*F*_(12, 1182)_ = 3.50, *p* < 0.001, $\eta_{p}^{2}$ = 0.03]. See Table 8.

**Table 8:** Mean scores and standard deviations in MLU3-w scale across age and input groups.

**Age**	**Input**	**Mean**	**Standard deviation**	***N***
18–24	Monolingual	1.94	1.26	200
	Basque-dominant bilingual	2.06	1.23	63
	Balanced bilingual	1.65	0.89	41
	Spanish-dominant bilingual	1.78	1.23	20
25–30	Monolingual	4.29 a	1.75	221
	Basque-dominant bilingual	3.84 ab	1.89	96
	Balanced bilingual	3.02 bc	1.97	35
	Spanish-dominant bilingual	2.29 c	1.49	19
31–36	Monolingual	5.64 a	2.43	107
	Basque-dominant bilingual	5.57 ab	2.41	26
	Balanced bilingual	4.23 abc	1.97	22
	Spanish-dominant bilingual	2.83 c	1.95	14
37–42	Monolingual	6.74 a	2.40	109
	Basque-dominant bilingual	6.36 a	2.77	26
	Balanced bilingual	5.92 a	3.62	20
	Spanish-dominant bilingual	3.76 b	2.31	24
43–48	Monolingual	7.45 a	2.49	102
	Basque-dominant bilingual	8.10 a	2.91	26
	Balanced bilingual	5.51 b	2.52	19
	Spanish-dominant bilingual	3.93 b	1.87	12

*Means that do not share a common alphabetical subscript differ at p < 0.05 (a > b > c) according to post hoc analyses with a Bonferroni correction*.

Further analyses on the interaction between input and age were performed by analyzing the effect of input in each age group by means of one-way ANOVAs. Regarding the analysis on MLU3-w (see Figure 6 and Table 8), no significant differences were observed across the four input groups in the youngest age group (between 18 and 24 months), [*F*_(3, 320)_ = 1.06, *p* = 0.364, $\eta_{p}^{2}$ = 0.01]. However, significant differences were observed across input groups above 2 years of age: for the 25- to 30-month-olds [*F*_(3, 367)_ = 11.18, *p* < 0.001, $\eta_{p}^{2}$ = 0.08], for the 31- to 36-month-olds [*F*_(3, 165)_ = 7.49, *p* < 0.001, $\eta_{p}^{2}$ = 0.12], for the 37- to 42-month-olds [*F*_(3, 175)_ = 8.72, *p* < 0.001, $\eta_{p}^{2}$ = 0.13] as well as for the 43- to 48-month-olds [*F*_(3, 155)_ = 10.80, *p* < 0.001, $\eta_{p}^{2}$ = 0.17]. Interestingly, the size of the input effect increased with age, reaching a large size from 3 years of age (37–42 months) onwards.

Similar results were also found in MLU3-m, with significant main effects of age [*F*_(4, 1182)_ = 108.25, *p* < 0.001, $\eta_{p}^{2}$ = 0.27] and input [*F*_(3, 1182)_ = 45.97, *p* < 0.001, $\eta_{p}^{2}$ = 0.10]. In addition, the interaction between age and input proved significant [*F*_(12, 1182)_ = 3.99, *p* < 0.001, $\eta_{p}^{2}$ = 0.04] (see Table 9).

**Table 9:** Mean scores and standard deviations in MLU3-m scale across age and input groups.

**Age**	**Input**	**Mean**	**Standard deviation**	***N***
18–24	Monolingual	2.52	1.77	200
	Basque-dominant bilingual	2.70	1.68	63
	Balanced bilingual	1.99	1.09	41
	Spanish-dominant bilingual	2.28	1.78	20
25–30	Monolingual	6.20 a	2.91	221
	Basque-dominant bilingual	5.51 ab	3.16	96
	Balanced bilingual	4.28 b	2.88	35
	Spanish-dominant bilingual	3.17 c	1.92	19
31–36	Monolingual	8.63 a	3.97	107
	Basque-dominant bilingual	8.25 a	4.00	26
	Balanced bilingual	6.25 a	3.42	22
	Spanish-dominant bilingual	4.12 b	3.14	14
37–42	Monolingual	10.59 a	3.91	109
	Basque-dominant bilingual	9.71 a	4.33	26
	Balanced bilingual	8.63 ab	5.37	20
	Spanish-dominant bilingual	5.48 b	3.78	24
43–48	Monolingual	11.84 a	4.11	102
	Basque-dominant bilingual	12.69 a	4.44	26
	Balanced bilingual	8.24 b	3.68	19
	Spanish-dominant bilingual	5.67 b	2.58	12

*Means that do not share a common alphabetical subscript differ at p < 0.05 (a > b > c) according post hoc analyses with a Bonferroni correction*.

Concerning MLU3-m (see Figure 6 and Table 9), no significant differences were observed among the four input groups in the youngest age range (18–24 months) [*F*_(3, 320)_ = 1.63, *p* = 0.182, $\eta_{p}^{2}$ = 0.01]. Nevertheless, from the age of 2 the effect of input in the MLU-w was revealed to be significant in all age groups: 25–30 months of age [*F*_(3, 367)_ = 9.73, *p* < 0.001, $\eta_{p}^{2}$ = 0.07], 31–36 months of age [*F*_(3, 165)_ = 7.19, *p* < 0.001, $\eta_{p}^{2}$ = 0.12], 37–42 months of age [*F*_(3, 175)_ = 10.37, *p* < 0.001, $\eta_{p}^{2}$ = 0.15] and 43–48 months of age [*F*_(3, 155)_ = 12.99, *p* < 0.001, $\eta_{p}^{2}$ = 0.20]. Similar to the pattern observed in MLU3-w, the size of the input effect increased with age, reaching a large size from age 3 onwards (37–42 m).

*Post hoc* analyses with a Bonferroni correction indicated no significant differences among input groups on MLU3-w and MLU3-m scores in the youngest age group (18–24 months). However, from 2 years of age, the mean scores for monolinguals and Basque-dominant bilinguals were significantly higher than those of the Spanish-dominant bilinguals (see Tables 8, 9). In contrast, monolinguals and Basque-dominant bilinguals did not differ significantly throughout the whole period studied, whilst balanced bilinguals showed intermediate scores which were closer to those of the Spanish-dominant bilingual group than to the Basque-dominant bilinguals in the age ranges before the 42nd month. Finally, in the oldest age group (43–48 months), the balanced bilinguals aligned with the Spanish-dominant bilinguals rather than with the Basque-dominant ones, as shown in Figure 6 and Tables 8, 9.

Therefore, three main results can be drawn from the analyses provided above:

1. Large age effects were attested in MLU-w and MLU-m as well as in the rest of the scales of the BDCI-2 and BCDI-3 instruments, and high correlations were observed between both MLU scales and the other scales tested.
2. The two MLU scales showed almost perfect correlations.
3. Input groups behaved similarly in the 18–24-month-old group, but differences among input groups started to be significant from age 2 onwards, in such a way that monolingual and Basque-dominant bilinguals differed more and more from the Spanish-dominant bilinguals with age, whereas the balanced bilingual group consistently showed intermediate MLU values between the groups with high (Basque-dominant) and with low (Spanish-dominant) exposure to the Basque language.

## Discussion

This paper is in line with previous research which used mean length of utterance, in general, and MLU3 in particular, as an accurate index of language development for individual assessment (Brown, 1973; Fenson et al., 1993, 2007). The present bilingual data further indicate that an appropriate use of the measurement which takes into account the amount of exposure to which children are exposed will favor a more accurate assessment of these children's actual language development.

The current study, which reported MLU data of Basque obtained by means of parental questionnaires from 16- to 50-month-olds, challenged general objections regarding the reliability (a) of parental reports to assess children's expressive language, (b) of MLU as an index for language development, and (c) the accuracy of measuring MLU in words in an agglutinative language with complex morphology.

Subjectivity is one of the strongest criticisms made regarding the CDI instrument in general and the MLU3 measure in particular. Nevertheless, many studies have defended the ecological validity of parental reports as compared to studies based on experimental data, based on the observation that parents witness their children's language use in manifold communicative situations (Institute of Medicine, 2001; American Academic of Pediatrics, 2003; O'Neil, 2007). Moreover, many handbooks of the adaptations of the CDI instruments to English and many other languages include validity studies comparing CDI parental report data with data obtained using other methodologies such as elicitation, or spontaneous interaction. These studies also reported strong correlations between MLU3 and the rest of the scales (Fenson et al., 1993; Jackson-Maldonado et al., 2003; López-Ornat et al., 2005; Barreña et al., 2008a). As for the subjectivity in coding MLU in general, and MLU3 in particular, the current study was based on data coded by two different researchers for both BCDI-2 and BCDI-3 data. The high correlation found between the two analyses confirmed the reliability of the coding used. The Basque sample data of 1337 children between 16 and 50 months of age obtained with either the BCDI-2 or the BCDI-3 revealed a gradual increase of mean scores in the scales studied throughout the age groups, month by month, similar to the one found in the lexical and grammatical scales of the BCDI-2 and BCDI-3. The high correlations found between MLU3-w, MLU3-m and the scales of vocabulary, verbal morphology, nominal morphology as well as with the section of multiple choice items regarding children's advance in the acquisition of some particular structures revealed an extremely strong internal consistency throughout the two parental questionnaires. Such a consistency proves, first, parental reports' trustworthiness when reporting about their children's language use and, second, BCDI instruments' reliability.

The first prediction—that MLU3 scales in BCDI would be as sensitive as the rest of the scales in this instrument in detecting toddlers' developmental changes—has been confirmed by the data analyzed. On the one hand, the large size of age effects on the BCDI scales tested confirmed the sensitivity of MLU-w and MLU-m as well as the rest of the scales in detecting developmental changes in both instruments ($\eta_{p}^{2}$ = 0.43 in BCDI-2, $\eta_{p}^{2}$ = 0.12–0.13 in BCDI-3). The effect size in the rest of scales was $\eta_{p}^{2}$ = 0.41–0.51 in BCDI-2 and lower, but still large or close to it ($\eta_{p}^{2}$ = 0.11–0.16) in BCDI-3. The fact that the effect size of age decreased from BDCI-2 ($\eta_{p}^{2}$ ≈ 0.40) to BCDI-3 ($\eta_{p}^{2}$ ≈ 0.15) can be explained in two ways. First, methodological differences such as the number of items included in the two instruments (see Table 1) may be the reason, at least partially, for the difference in the effect of age: the differences in the number of items are large in vocabulary (643/120 words). However, they are not so big in morphology (17/16 in nominal morphology and 40/20 in verbal morphology) where, nevertheless, the effect size of age decreased at the same pace as for vocabulary. Moreover, MLU scales were calculated in exactly the same way in both instruments and revealed again a weaker effect of age in BCDI-3 than in BDCI-2, questioning the relevance of the methodological account for the differences mentioned. The second explanation in terms of development appears to be much more convincing: the difference attested between the two Basque instruments is compatible with the stronger developmental changes taking place between the earlier developmental period covered by the BCDI-2 (16–30 months), as compared to the later one covered by the BDCI-3 (30–50 months). The decrease in developmental speed found in the Basque data is in line with that found by Fenson et al. (2007) with the English instruments CDI-2 (16–30 m) and CDI-3 (30–42 m), and with Brown's statement that MLU scales may not be accurate enough for measuring language complexity once the child has reached Stage V. Note that two of the children studied by Brown reached that stage at around age 4, whilst the third one had reached it almost 2 years earlier. Hence, this is compatible with the idea that the effect of this factor decreases after some age between 3 and 4 years.

On the other hand, the high correlations between MLU and the rest of the scales reveals the consistency of the instrument and its validity to measure children's verbal communicative development between 16 and 50 months of age in line with the results of many adaptations of the CDI-2 and CDI-3 instruments (Fenson et al., 1993, 2007; Jackson-Maldonado et al., 2003; López-Ornat et al., 2005). Even though the explanation is not clearly formulated yet, we can conclude, in line with Dethorne et al. (2005), that the strong correlation attested between MLU values and scales of varied instruments used across studies to measure children's development in different language components (expressive vocabulary, grammar…) confirms Brown's assumption that MLU is a measure of early development in language complexity in general, rather than of a specific language component, such as semantics or morphosyntax, in particular. Its validity may be limited to the earliest stages, applying no further than Stage V. Nonetheless, this last point could not be either confirmed or disconfirmed by the Basque data and requires further research.

The second hypothesis that MLU3-m would turn out to be more discriminative than MLU-w has not been confirmed by the data, since no size differences were found in the effect of age in the two MLU scales: $\eta_{p}^{2}\;=$ 0.43 in BCDI-2 and $\eta_{p}^{2}$ ≈ 0.11 in BDCI-3. Moreover, the almost perfect correlations between the two MLU scales indicate their similar validity to measure utterance length, regardless of the specific unit (word/morpheme) adopted as baseline. Based on the high correlations found in studies comparing MLU-w and MLU-m scores in several languages (and even MLU counted in syllables), many authors consider that both MLU measures function equally well for measuring grammatical development (Hickey, 1991; Aguado, 1995; Parker and Brorson, 2005). In contrast, Wieczorek (2010) considers that each MLU scale measures development in a different language component: MLU-w being more related to lexical development, and MLU-m to morphological development. Our data support the former position. The high correlations between the two scales in both instruments (*r* > 0.97 and *r* > 0.95, when age is controlled) confirm the utility of both indexes to measure development in language complexity. Moreover, regardless of measuring MLU3 in words or in morphemes, correlations between MLU3-m and the rest of the scales are almost identical to those between MLU3-w and the same scales, regardless of the lexical or grammatical character of them, in contrast to what has been suggested by Wieczorek (2010). The relations across MLU measurements and between MLU3-w and MLU3-m and the rest of scales may vary across languages or language types which differ in degree of morphological complexity and transparency (agglutinative, fusionant, polysynthetic…), but such an analysis goes far beyond the scope of the current paper.

Utterance segmentation in words is much quicker and easier, since no technical descriptions are necessary, fewer decisions are required (less subjectivity) and variability across coders decreases considerably, in line with previous studies (Hickey, 1991; Jackson-Maldonado and Conboy, 2007, among others). The redundancy of using both, in addition to the ease of segmenting the utterance in words as compared to morphemes, leads us to recommend MLU-w as a more parsimonious measurement for screening in clinical studies, as has been suggested in other languages (Hickey, 1991; Parker and Brorson, 2005), without denying MLU-m's utility for more specific surveys in research.

The third hypothesis, that the relative amount of input would affect children's MLU, has been partially confirmed. MLU3 scales proved sensitive to detect input effects. A subsample of around 1200 children aged 18–48 months was analyzed with more detail in order to test MLU3's utility to test children's attained developmental level in the acquisition of a minority language in permanent contact with another socially dominant Romance language (Spanish or French). The data revealed MLU3-w and MLU3-m's sensitivity not only to age, already tested in Basque as in many other languages, but to the relative amount of exposure to the language. However, the effect of the amount of (relative) exposure to the language was not visible in the youngest child group (18–24 months). Interestingly, the effect of input increased with age after age 2, varying from medium at age 2 ($\eta_{p}^{2}$ = 0.07 and 0.12) to large at age 3 ($\eta_{p}^{2}\;=$ 0.15 and 0.20). From age 2 onwards, children with a large amount of exposure to Basque (M and BDB groups) showed more similar scores in MLU3-w and MLU3-m scales than the group with less exposure (SDB), in line with previous studies which tested these populations' lexical and grammatical scores (Barreña et al., 2008a,b).

Despite the strong intralinguistic correlations found among the BCDI subscales, in line with CDI data of English-Spanish bilinguals (Marchman et al., 2004; Hoff et al., 2014), measuring Basque bilinguals' language use only in Basque leads us to under-score the real language capacity of most participants in the present study. Children who are exposed to more than one language rarely have the same amount of exposure to one of the languages as compared to age-matched monolinguals, on whom normative data are based (Ezeizabarrena et al., 2017). As has been shown very convincingly by Pearson et al. (1997), bilingual assessment should ideally take place in their two languages, and in this vein, the accurate evaluation of Basque-Spanish bilinguals' communicative skills should include assessing MLU in their two languages.

## Conclusions

The analysis of cross-sectional data obtained with the BCDI-2 (16–30 months) and BCDI-3 (30–50 months) of over 1200 children revealed a strong correlation between MLU3 and expressive vocabulary in both instruments, as well as between MLU3 and morphological scales. These findings confirm the consistency of the MLU measurement, as well as that of both BCDI instruments. The results also showed that MLU3-w and MLU3-m scales can report equally well on very young children's development in the Basque language up to age 4, which leads us to recommend the easier MLU-w measurement for clinical purposes. Finally, MLU3 subscales proved sensitive to input (25–48 months), which indicates the utility of these subscales to identify developmental patterns in Basque bilinguals aged 2–4.

## Ethics statement

This study was approved by the ethics commission of the University of the Basque Country.

## Author contributions

Data-analysis: IG and M-JE. Manuscript writing and editing: M-JE and IG.

### Conflict of interest statement

The authors declare that the research was conducted in the absence of any commercial or financial relationships that could be construed as a potential conflict of interest.

## Footnotes

^1^Only the very few instances of -*txo* attached to words other than *ama, amatxo* “mom, mommy” and *aita, aitatxo* “dad, daddy” were counted as morphemes.

^2^Correlation between MLU and the scores obtained in the multiple choice question section in BCDI-2 yielded statistically significant results (*p* < 0.001): MLU3-w (*r* = 0.77 and *r* = 0.64, controlling for age) and MLU3-m (*r* = 0.77 and *r* = 0.63, controlling for age). The analysis of the multiple choice item sections goes beyond the purpose of the current study. Nonetheless, we have reported these data because of the request of one anonymous reviewer.

^3^MLU and the scores obtained in the multiple choice question section in BCDI-3 also yielded statistically significant results (*p* < 0.001): MLU3-w (*r* = 0.60 and *r* = 0.54, controlling for age) and MLU3-m (*r* = 0.64 and *r* = 0.59, controlling for age).
